# A Probabilistically Weakly Secure Network Coding Scheme in Multipath Routing for WSNs

**DOI:** 10.3390/s17051133

**Published:** 2017-05-16

**Authors:** Xiang Liu, Jie Huang, Xiang Gao

**Affiliations:** School of Information Science and Engineering, Southeast University, Nanjing 210096, China; jhuang@seu.edu.cn (J.H.); xianggao@seu.edu.cn (X.G.)

**Keywords:** probability of transmission being secure, weakly secure network coding, multipath routing, energy efficiency

## Abstract

In wireless sensor networks, nodes are mostly deployed in unsupervised areas and are vulnerable to a variety of attacks. Therefore, data security is a vital aspect to be considered. However, due to the limited computation capability and memory of sensor nodes, it is difficult to perform the complex encryption algorithm, as well as the key distribution and management algorithm. Toward this end, a low-complexity algorithm for security in wireless sensor networks is of significant importance. In this article, a weakly secure network coding based multipath routing scheme is proposed, which can guarantee the data confidentiality in transmission probabilistically, and can improve the energy efficiency in the meantime. Then the simulations of the probability of transmission being secure are performed. The results show that with the increase of the number of hops *k*, the probability of transmission being secure suffers from a rapid decrease. On the contrary, with the increase of multicast capacity *h* it undergoes a slight growth. Therefore, the weak security can be achieved with probability approaching 1 by limiting the number of hops and increasing the multicast capacity. Meanwhile, the simulations of energy consumption are performed and the comparison between the energy consumption of the scheme in this article and the multipath routing scheme without network coding is conducted. The results show that by employing network coding, the scheme in this article can improve the energy efficiency, and the more packets transmitted, the more energy consumption can be reduced.

## 1. Introduction

Due to the complex working environment, wireless sensor networks (WSNs) can suffer from a variety of attacks. Therefore, transmission security, including data confidentiality, data integrity, and data availability, is a vital aspect to be considered [1,2,3,4]. Existing researches on security of WSNs are mostly based on encryption/decryption. In [5], a symmetric encryption algorithm is proposed, which is an amalgamation of two different encryption algorithms in randomized method. In [6], the authors analyzed the security challenges in WSNs and smart home systems, then proposed a security evaluation technique based on attack graph generation. SNEP protocol is one of most maturely applied security protocols in WSNs [7]. Since the communication among the nodes requires the involvement of the base station, the SNEP protocol is of rather low efficiency and is not applicable in large-scale networks. Since the low-cost wireless sensors which are battery-powered have limited computational capability and memory, it is difficult to perform complicated encryption algorithms in WSNs. Moreover, achieving key distribution and key management is also a great challenge in large-scale WSNs. In [8], a random key distribution based key management protocol is proposed. In this protocol, a key pool with size of *S* is established firstly and each node in the network stores *m* keys of the key pool. Once any two nodes in the network have the same key, a secure channel can be established between them. However, the biggest problem is that if there are no identical keys between a pair of nodes, the secure data transmission between them cannot be completed. To overcome the disadvantages in the aforementioned techniques, a weakly-secure network coding based multipath routing scheme is proposed in this article to guarantee the confidentiality of messages. The superiority of the scheme in this article over encryption is that it gets rid of the complex encryption algorithm and key distribution/management, so it can greatly reduce the computation overhead.

Compared with Shannon security [9,10,11,12,13], the requirement of weak security in this article is not information-theoretically perfect [14]. While the Shannon security requires the attacker cannot get any information about the source message, in the paradigm of weak security, the attacker cannot get any useful information about the source message, which means the attacker cannot decode any part of the source message correctly. As a result, weak security can bring higher transmission capacity than Shannon security. Therefore, the weakly secure network coding based scheme is adopted in this article to improve the transmission efficiency and reduce the transmission overhead.

The main contributions of this article can be listed as follows. Firstly, the relationship between the probability of transmission being secure and some network parameters is presented through mathematical derivations and simulations. Then it is shown that by setting the parameters appropriately, the weak security can be achieved with the probability approaching 1. Secondly, by employing network coding in the intermediate nodes and calculating the least number of communication nodes to satisfy the network requirements, the method proposed in this article can largely reduce the transmission overhead compared with the multipath routing scheme without network coding.

The remainder of this article is organized as follows. In Section 2, the network model is introduced, including the adversary model and the algorithms to get the number of paths as well as the least number of communication nodes. Then the simulation results about these algorithms are presented as well. In Section 3, the pre-coding scheme and random network coding scheme are presented. In Section 4, the security analysis is conducted, and the probability of the transmission being weakly secure is derived. In Section 5, the analysis of energy consumption of two different schemes is performed, namely, the network coding based multipath routing scheme and the multipath routing scheme without network coding. In Section 6, the simulations based on the above analysis are performed and the results are presented. In Section 7, the conclusions based on the simulations are presented.

## 2. Network Model

### 2.1. Adversary Model

In this article, the communication network can be described as a directed acyclic graph G=(V,E), where *V* is the set of nodes and *E* is the set of links. For each link e∈E, we define tail(e) and head(e) as the tail and head of *e*, respectively. In the set of nodes *V*, it is denoted by *s* the source node, and by *T* the sink node, which is the base station in the practical WSNs. For each node v∈V, let Out(v) and In(v) denote the set of outgoing channels and incoming channels of *v*, respectively. That is, Out(v)={(v,u):(v,u)∈E} and In(v)={(u,v):(u,v)∈E}. For the eavesdropper, let $V_{eav}$ and $E_{eav}$ denote the set of nodes and channels being eavesdropped, respectively. The multi-cast capacity *H* is the minimum number of edges in any cut between the source node and sink node. Each channel e∈E contains a message packet, whose elements are selected from a finite field $F_{q}$, where *q* is the size of the finite field. For the source node, we introduce *H* artificial channels which carry the *H* source packets that the source node transmits to the base station.

It is supposed that there exist some nodes that are randomly deployed in a specified district, and the number of nodes is denoted by *N*. After the routing procedure is completed, there exist $N_{c}$ intermediate nodes that are involved in transmission. It is assumed that there is an adversary which is randomly located in the district, and for each intermediate node, there is a probability that it may be attacked by the adversary and the figure of the probability is up to the range that the adversary can control. Once the intermediate node is attacked, it can be controlled by the adversary and the messages it receives and transmits can be observed completely by the adversary. For each transmission, whether an individual node is controlled is independent. Notably, the source node and the base station cannot be attacked, otherwise the malicious node can get the message without any loss.

### 2.2. Calculation of Number of Paths

Suppose that the successful delivery ratio (SDR) is denoted by *r*. For every single link between any two nodes, the link failure probability is *e*. In addition, the average number of hops of the paths from the source node to the base station is *k*, the desired multicast capacity is *h*. To simplify the question, it is assumed that the number of hops of each path exactly equals to *k*. Transmission of packets on each hop can be regarded as dependent event, hence, for each path, the probability of successfully delivering one packet is

(1) $$p_{k}={\left(1-e\right)}^{k}$$

For one transmission, only if the number of successful paths is at least *h* do we call it a successful transmission. Since the desired multicast capacity is *h*, it requires H≥h paths to guarantee the expected successful delivery ratio *R*. Under the condition that link failure probability is *e* and the number of hops is *k*, let $H_{h,e,k,R}$ denote the least number of paths to implement to achieve capacity of *h* and SDR of *R*. Among the *H* paths, the number of successful paths should be at least *h* to guarantee the correct recovery of original message at the base station. Hence, the SDR can be represented as

(2) $$r=\sum_{i=h}^{H}C_{H}^{i}p_{k}^{i}{\left(1-p_{k}\right)}^{H-i}$$

where $C_{H}^{i}$ is the binomial coefficient, defined as $C_{H}^{i}=\frac{H!}{(H-i)!(i)!}$.

In this article, it is essential to determine the least number of *H*, referring to as $H_{h,e,k,R}$, to guarantee that the successful delivery ratio satisfies r≥R , i.e.

(3) $$\sum_{i=h}^{H_{h,e,k,R}}C_{H_{h,e,k,R}}^{i}p_{k}^{i}{\left(1-p_{k}\right)}^{H_{h,e,k,R}-i}\geq R$$

In practice, it can be impossible to find the analytical solution of formula (3). So we need to find the numeric solution by the iteration algorithm. However, when *h* gets larger, it can be of great computational complexity to perform the iteration algorithm to get *H*. Consequently, it is necessary to adopt another algorithm with light complexity to get the approximated solution of (3), which is also presented in [15,16]. Let $H_{s}$ denote the number of the successful paths in the *H* paths, then $H_{s}$ satisfies the Binomial distribution B(*H*,$p_{k}$), and the mean value and the variance of $H_{s}$ can be written as

(4) $$\mu =E\left(H_{s}\right)=Hp_{k}=H{\left(1-e\right)}^{k}$$

(5) $$\sigma^{2}=Hp_{k}\left(1-p_{k}\right)=H{\left(1-e\right)}^{k}\left(1-{\left(1-e\right)}^{k}\right)$$

The successful delivery ratio *r* can be rewritten as

(6) $$r=P(H_{s}\geq h)$$

Thus, the question can be described as finding the least *H* that guarantees $P(H_{s}\geq h)\geq R$. According to the central-limit theorem, the Binomial distribution can be regarded as Normal distribution approximately, i.e.,

(7) $$H_{s}∼N\left(\mu ,\sigma^{2}\right)$$

Let

(8) $$H_{s}^{*}=\frac{H_{s}-\mu }{\sigma }$$

then $H_{s}^{*}$ satisfies the standard normal distribution, i.e.

(9) $$H_{s}^{*}∼N\left(0,1\right)$$

In the standard normal distribution, for the given *R*, the value of $x_{R}$ that satisfies $P(H_{s}^{*}\geq x_{R})\geq R$ can be obtained from the probability density function of standard normal distribution, which means

(10) $$h=x_{R}\sigma +\mu$$

After that, the value of *H* can be calculated through the Equation (11).

(11) $$h=x_{R}\sigma +\mu =x_{R}\sqrt{H{\left(1-e\right)}^{k}\left(1-{\left(1-e\right)}^{k}\right)}+H{\left(1-e\right)}^{k}$$

Hereto, given the desired successful delivery ration *R*, the link failure probability *e*, the average number of hops *k*, and the expected multi-cast capacity *h*, the least number of paths can be calculated according to Algorithm 1:
**Algorithm 1** approximated algorithm of Calculating *H*1:Initializing with *R*, *e*, *k*, and *h*2:Calculating $x_{R}$3:Calculating *H*

**Figure 1:**
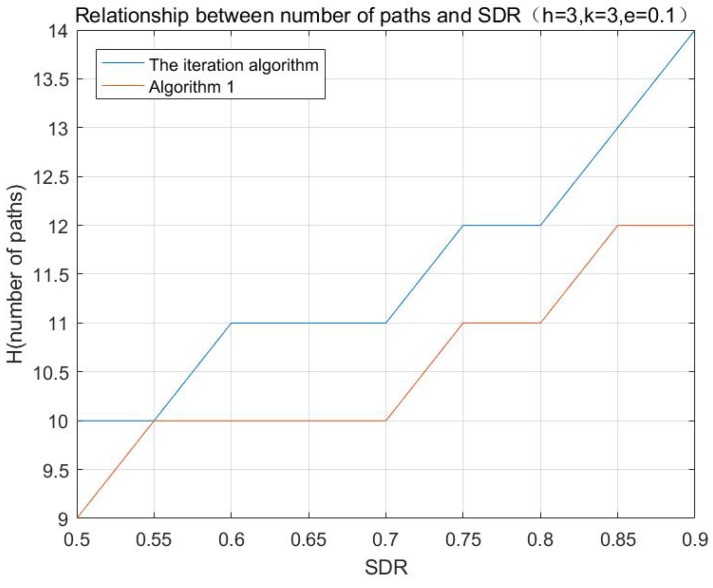
Relationship between number of paths and SDR.

**Figure 2:**
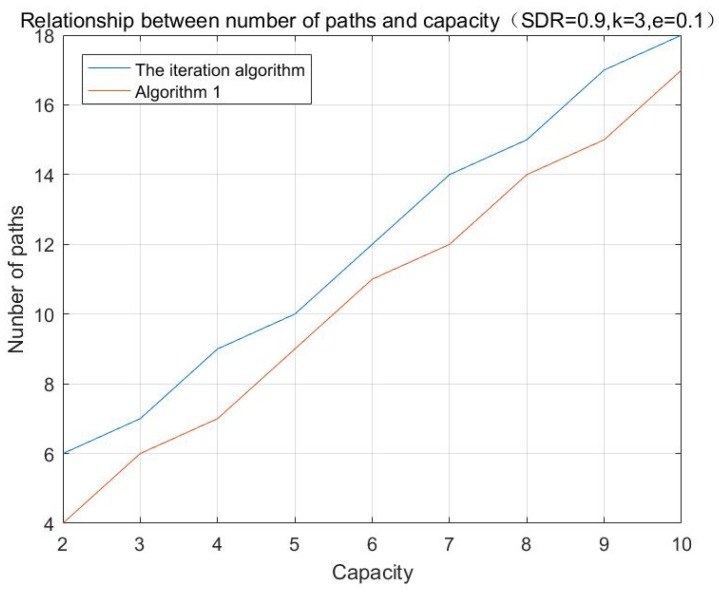
Relationship between number of paths and multi-cast capacity.

**Figure 3:**
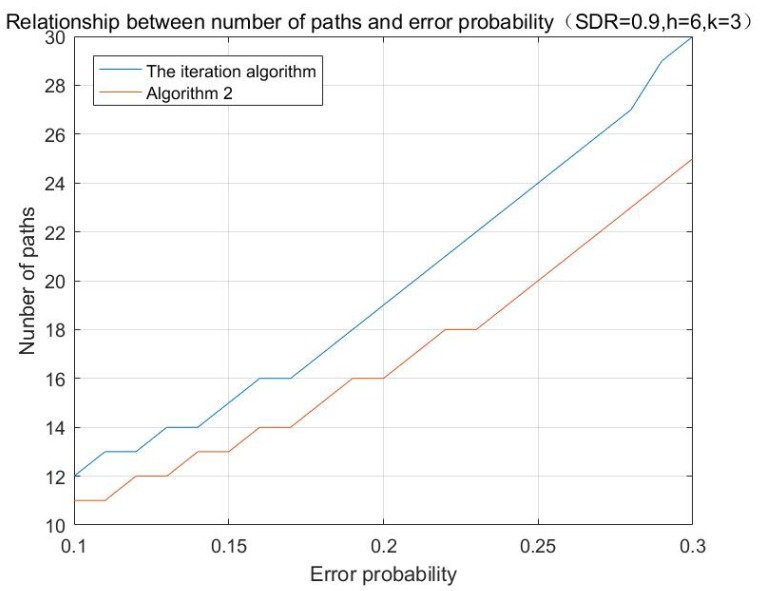
Relationship between number of paths and error probability.

According to the aforementioned algorithms, the simulations with different initializing values are performed, and the results are shown in Figure 1, Figure 2 and Figure 3:

### 2.3. Least Number of Communication Nodes

After getting the least number of paths *H* according to the algorithms mentioned above, the routing procedure can be proceeded to establish those *H* paths to build the communication network. For the sake of energy efficiency and transmission security, it is essential to involve as few nodes as possible in communication under the network conditions. Given the number of hops of each path, and the number of paths, we define $N_{l}$ as the least number of communication nodes, a.k.a the least number of nodes that need to be involved in communication to satisfy the conditions.

**Algorithm 2** The algorithm to calculate the least number of communication nodes1:Initiate with the parameters H,k2:Create the first path and set num_node←k-1,s_path←13:**while** s_path<H **do**4:     \\ **Create a new path**5:     p_flag←06:     **while** p_flag==0 **do**7:          c_hop←08:          **while** c_hop<k-1 **do**9:                **for** i=1:num_node **do**10:                      new_node←111:                       **if** the i-th node is the current hop||exists parallel channels||exists a loop **then**12:                             Continue13:                       **else**14:                             pick the i-th node as the next hop15:                             new_node←016:                             break17:                       **end if**18:                **end for**19:                **if** new_node==1 **then**20:                      pick up a new node as the next hop21:                       num_node←num_node+122:                **end if**23:                c_hop←c_hop+124:          **end while**25:          p_flag←126:          s_path←s_path+127:     **end while**28:**end while**29:$N_{l}\leftarrow num\_node$

In Algorithm 2, the first path with *k* hops is established initially. To do so, there should be k-1 intermediate nodes involved. Then, it can be proceeded to establish the rest H-1 paths. What is notable is that the network topology is node-braided and edge-disjoint, which means that between any two paths, there may exist common nodes but cannot exist any common edges. Therefore, when executing the routing, it should be firstly determined whether the existing intermediate nodes can be used as the next hop of the current path, if not, then it is necessary to introduce a new node. Meanwhile, it is crucial that there cannot exist parallel links between any two nodes, either loops in the whole network.

**Figure 4:**
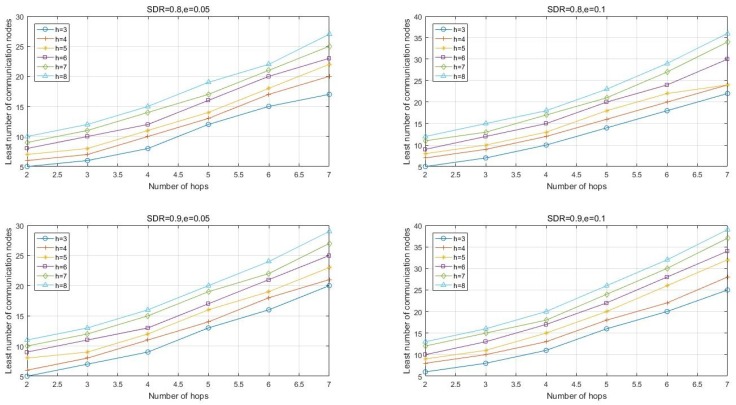
The least number of communication nodes.

According to Algorithm 2, some simulations are performed and the results are shown in Figure 4.

## 3. Weakly Secure Network Coding

**Figure 5:**
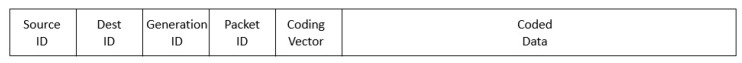
Packet Format.

In this section, a packet format which can get rid of centralized knowledge of network topology is proposed, which is shown in Figure 5.

Here the ’Source ID’ is the ID of the transmitting node, the ’Dest ID’ is the ID of the receiving node, and the ’Generation ID’ is the identifier of a generation. The source node categories all the source packets into some groups and each group includes *h* source packets. Such a group is called one generation. In each generation, the *h* packets are assigned with a packet ID ranging from 1 to *h* respectively. In addition, the source sends one generation in each transmission. The ’Coding Vector’ is the vector of combination coefficients of the coded packet.

To achieve weak security, the source node needs to encode the data before transmitting it and this process is called pre-coding. The pre-coding algorithm in this article is generated from the algorithm in [17]. Compared with the algorithm in [17], the algorithm in this article introduces more non-linear property to the coded packets.

According to the hypothesis, the source node can transmit *h* packets in one transmission. Therefore, without loss of generality, the source message can be denoted as

(12) $$M={\left[m_{1},m_{2},\cdots ,m_{h}\right]}^{T}$$

Then the pre-coded message can be written as

(13) $$M^{{}^{\prime }}={\left[m_{1}^{{}^{\prime }},m_{2}^{{}^{\prime }},\cdots ,m_{h}^{{}^{\prime }}\right]}^{T}$$

where

(14) $$\left\{\begin{array}{c} m_{1}^{{}^{\prime }}=f\left(m_{1}\right)+m_{2}\\ m_{2}^{{}^{\prime }}=f\left(m_{1}+m_{2}\right)+m_{3}\\ \vdots \\ m_{n-1}^{{}^{\prime }}=f\left(m_{1}+m_{2}+\cdots +m_{n-1}\right)+m_{n}\\ m_{n}^{{}^{\prime }}=m_{1}+f\left(m_{1}^{{}^{\prime }}+\cdots +m_{n-1}^{{}^{\prime }}\right) \end{array}\right.$$

The function *f* is a permutation function and both its input and output are vectors which consist of elements in the finite field. Note that the construction of function *f* is public to all nodes, even including the adversary.

After the pre-coding procedure is completed, the source node applies the generating matrix G to the coded message to generate *H* packets to transmit along the *H* outgoing channels of the source node *s*,

(15) $$X=GM^{{}^{\prime }}={\left[x_{1},\cdots ,x_{H}\right]}^{T}$$

where G is a *H*-by-*h* matrix whose elements are chosen randomly from the finite field.

For the sink node, the received packets can be denoted as $Y={\left[y_{1},y_{2},\cdots ,y_{m}\right]}^{T}=CM^{\prime }$, where C is the coding matrix of Y, and the i-th row of C is the coding vector of packet $y_{i}$. Then by using Gaussian elimination method, $M^{\prime }$ can be calculated. After that, $m_{1},m_{2},\cdots ,m_{h}$ can be calculated iteratively according to formula (16):

(16) $$\left\{\begin{array}{c} m_{1}=m_{n}^{{}^{\prime }}-f\left(m_{1}^{{}^{\prime }}+\cdots +m_{n-1}^{{}^{\prime }}\right)\\ m_{2}=m_{1}^{{}^{\prime }}-f\left(m_{1}\right)\\ \vdots \\ m_{n-1}=m_{n-2}^{{}^{\prime }}-f\left(m_{1}+\cdots +m_{n-2}\right)\\ m_{n}=m_{n-1}^{{}^{\prime }}-f\left(m_{1}+m_{2}+\cdots +m_{n-1}\right) \end{array}\right.$$

In this way, the sink node can decode all the *h* packets in one generation.

## 4. Security Analysis

Since each node is deployed randomly in the district and the adversary is randomly located in the district, then for every single node, the probability of being located in the overhearing zone (being controlled by the adversary) is

(17) $$p_{o}=\frac{S_{overhear}}{S_{total}}$$

where $S_{total}$ is the size of the whole district wherein the wireless sensors are deployed, and $S_{overhear}$ is range that the adversary can control. Once a node is located in that range, it will be controlled by the adversary. Therefore, in the whole district, the number of nodes which are overheard $N_{o}$ satisfies the binomial distribution

(18) $$N_{o}∼B\left(N,p_{o}\right)$$

Given the average number of hops of each path and the number of paths, define $N_{co}$ as the number of communication nodes which be overheard by the attacker.

**Theorem** **1.** *The probability of $N_{co}=m$ for all integers $0\leq m\leq N_{l}$ can be denoted as*

(19) $$p\left(N_{co}=m\right)=C_{N}^{N_{co}}{\left(p_{c}p_{o}\right)}^{N_{co}}{\left(1-p_{c}p_{o}\right)}^{N-N_{co}}$$

*where $p_{c}=\frac{N_{l}}{N}$ is the probability of a node being involved in the communication.*

**Proof.**  (20) $$\begin{array}{cc} p(N_{co}=m) & =\sum_{k=m}^{N}p\left(N_{o}=k\right)p\left(N_{co}=m|N_{o}=k\right)\\ & =\sum_{k=m}^{N}C_{k}^{N_{co}}p_{c}^{N_{co}}{\left(1-p_{c}\right)}^{k-N_{co}}{\left(1-p_{c}\right)}^{N-k}C_{N}^{k}p_{o}^{k}{\left(1-p_{o}\right)}^{N-k}\\ & =C_{N}^{N_{co}}{\left(p_{c}p_{o}\right)}^{N_{co}}{\left(1-p_{c}p_{o}\right)}^{N-N_{co}} \end{array}$$

 ☐

**Theorem** **2.** *Let $E_{o}$ denote the number of channels that being overheard by the attacker, and $E_{vo}$ denote the number of valid channels that being overheard, then for all integers 0≤c≤kH*

(21) $$\begin{array}{cc} p(E_{vo}=c) & =\sum_{i=c}^{kH}p\left(E_{o}=i\right)p\left(E_{vo}=c|E_{o}=i\right)\\ & =\sum_{i=c}^{kH}p\left(E_{o}=i\right){\left(1-e\right)}^{c}e^{\left(i-c\right)} \end{array}$$

And $p\left(E_{o}=i\right)=\sum_{j=0}^{N_{l}}p\left(N_{co}=j\right)p\left(E_{o}=i|N_{co}=j\right)$, so Equation (27) can be rewritten as

(22) $$p\left(E_{vo}=c\right)=\sum_{i=c}^{kH}\sum_{j=0}^{N_{l}}p\left(N_{co}=j\right)p\left(E_{o}=i|N_{co}=j\right){\left(1-e\right)}^{c}e^{\left(i-c\right)}$$

Let $\Gamma_{w}$ be the overhearing matrix, referring to as the matrix that consists of the coding vectors of the valid channels being overheard.

**Theorem** **3.** The attacker cannot get any useful information of the original messages given that $R(\Gamma_{w})<h$, where $R(\Gamma_{w})$ is the rank of matrix $\Gamma_{w}$, i.e., $\Gamma_{w}$ is not a full-rank matrix.

**Proof.**  Let $X={\left(x_{1},x_{2},\cdots ,x_{h}\right)}^{T}$ be the original message that is sent over the network. Then after the pre-coding on the source node, the input message can be written as $X^{\prime }=\left[x_{1}^{\prime },x_{2}^{\prime },\cdots ,x_{h}^{\prime }\right]$, i.e., the source transmits $X^{\prime }$ instead of X. Since a linear random network code is used ,the message on each channel $e_{j}$ can be written as $\Gamma_{e_{j}}X^{\prime }$. The message obtained by the attacker is **W**=$\Gamma_{w}X^{\prime }$. Since $R(\Gamma_{w})<h$, which means the adversary can obtain at most h-1 linearly independent equations, which means that it cannot resolve for all the packets in $X^{\prime }$. Then the attacker cannot solve any packets through formula (16). Hence, we have $I\left(x_{i};B\right)=I\left(x_{i};W\right)=0$ and by so we can achieve weak security.  ☐

Let $p_{e}$ be the probability of transmission being insecure, which means

(23) $$p_{e}=p\left(R\left(\Gamma_{w}\right)=h\right)$$

Then $p_{s}=1-p_{e}$ is the probability of transmission being secure.

**Theorem** **4.** (24) $$\begin{array}{cc} p_{e} & =\sum_{m=0}^{kH}p\left(E_{vo}=m\right)p\left(R\left(\Gamma_{w}\right)=h|E_{vo}=m\right)\\ & =\sum_{m=h}^{kH}p\left(E_{vo}=m\right)p\left(R\left(\Gamma_{w}\right)=h|E_{vo}=m\right) \end{array}$$

**Proof.**  When $E_{vo}\leq h-1$, it is obvious that the rank of overhearing matrix $\Gamma_{w}$ cannot be *h* since the maximum value of $R(\Gamma_{w})$ is $E_{vo}\leq h-1$. Hence, for 0≤m≤h-1, $p(R\left(\Gamma_{w}\right)=h|E_{vo}=m)=0$. When h≤m≤kH, the probability of R(C)=h under the condition of $E_{vo}=m$ is $p(R\left(\Gamma_{w}\right)=h|E_{vo}=m)$. In summary, $p_{e}=\sum_{m=h}^{kH}p\left(E_{vo}=m\right)p\left(R\left(\Gamma_{w}\right)=h|E_{vo}=m\right)$, then Theorem 4 proved. ☐

**Lemma** **1.** *For h≤m≤kH, we have*

(25) $$p(R\left(\Gamma_{w}=h|E_{vo}=m\right)=\prod_{i=0}^{h-1}\left(1-q^{i-m}\right)$$

*where q is the size of the finite field.*

**Proof.**  Let $N_{m,h}\left(h\right)$ denote the number of m-by-h matrices that have a rank of *h*(m≥h). Then form [18], we have

(26) $$\begin{array}{cc} N_{m,h}\left(h\right) & =q^{mh}\prod_{i=0}^{h-1}\frac{\left(1-q^{i-h}\right)\left(1-q^{i-m}\right)}{\left(1-q^{i-h}\right)}\\ & =q^{mh}\prod_{i=0}^{h-1}\left(1-q^{i-m}\right) \end{array}$$

Hence, we have

(27) $$\begin{array}{cc} p(R\left(\Gamma_{w}\right)=h|E_{vo}=m) & =\frac{N_{m,h}\left(h\right)}{q^{mh}}\\ & =\prod_{i=0}^{h-1}\left(1-q^{i-m}\right) \end{array}$$

This, we complete the proof of Lemma 1. ☐

**Theorem** **5.** *According to Lemma 1, we have*

(28) $$\begin{array}{cc} p_{e} & =\sum_{m=h}^{kH}p\left(E_{vo}=m\right)p\left(R\left(\Gamma_{w}\right)=h|E_{vo}=m\right)\\ & =\sum_{m=h}^{kH}p\left(E_{vo}=m\right)\prod_{i=0}^{h-1}\left(1-q^{i-m}\right) \end{array}$$

*Hence, the probability of transmission being secure can be written as*

(29) $$\begin{array}{cc} p_{s} & =1-p_{e}\\ & =1-\sum_{m=h}^{kH}p\left(E_{vo}=m\right)\prod_{i=0}^{h-1}\left(1-q^{i-m}\right) \end{array}$$

## 5. Power Consumption Analysis

**Figure 6:**
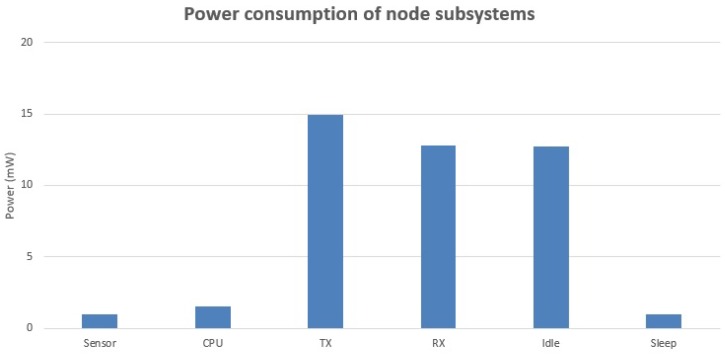
Power consumption of different components in wireless sensor nodes.

Figure 6 shows the power consumption of different components in WSNs, which is proposed by Estrin [19]. It indicates that compared with the energy consumed by data transmitting and receiving, the energy consumed by other components, including sensing, computing and sleeping can be negligible. Meanwhile, the energy consumption of idling is always allocated by the nodes to avoid collisions and does not affect the energy analysis in network layer since avoiding collisions is a function in MAC layer [20]. Therefore, in this article, the total energy consumption of one transmission can be written as

(30) $$E=E_{TX}+E_{RX}$$

Specifically, in one transmission, the energy consumption of transmitting and receiving can be written as

(31) $$E_{TX}\left(B,d\right)=E_{TXElec}*B+E_{amp}*B*d^{\gamma }$$

(32) $$E_{RX}\left(B,d\right)=E_{RXElec}*B$$

where *B* is the number of bits of data, $E_{TXElec}$ and $E_{RXElec}$ are the energy consumption of transmitting and receiving a bit of data respectively. In addition, $E_{amp}$ is the amplification factor of the amplifier, *d* is the distance between transmitting node and receiving node, γ is the path-loss factor. Therefore, when a source node needs to transmit a packet of *B* bits to a sink node through a path of *k* hops, the total energy consumption is

(33) $$\begin{array}{cc} E_{B,d,k} & =E_{TX}\left(B,d\right)*k+E_{RX}\left(B,d\right)*k\\ & =k*B*\left(E_{TXElec}+E_{RXElec}\right)+k*E_{amp}*B*d^{\gamma } \end{array}$$

In multipath routing scheme without network coding, to achieve a desired successful delivery ratio of *R*, the number of paths should be employed is

(34) $$P=\lceil \frac{\log(1-R)}{\log(1-{\left(1-e\right)}^{k})}\rceil$$

Therefore, to transmit $N_{p}$ packets successfully from the source to the node, the number of packets that source node needs to transmit totally is

(35) $$N_{total}^{1}=N_{p}*\left\lceil \frac{\log(1-R)}{\log(1-{\left(1-e\right)}^{k})}\right\rceil$$

Hence, the total energy consumption is

(36) $$E_{h,d,k}^{1}=k*N_{p}*\left\lceil \frac{\log(1-R)}{\log(1-{\left(1-e\right)}^{k})}\right\rceil *b*\left(E_{TXElec}+E_{RXElec}\right)+k*E_{amp}*N_{p}*\left\lceil \frac{\log(1-R)}{\log(1-{\left(1-e\right)}^{k})}\right\rceil *b*d^{\gamma }$$

where *b* is the number of bits per packet. On the other hand, in the network coding based multipath routing scheme, to achieve a desired successful delivery ratio of *R* with multicast capacity of *h*, the number of path needs to be employed *H* can be calculated by Algorithm 1. Then to transmit $N_{p}$ packets successfully from the source to the node, the number of packets that source node needs to transmit totally is

(37) $$N_{total}^{2}=N_{p}*\frac{H}{h};$$

Hence, the total energy consumption is

(38) $$E_{h,d,k}^{2}=k*N_{p}*\frac{H}{h}*b*\left(E_{TXElec}+E_{RXElec}\right)+k*E_{amp}*N_{p}*\frac{H}{h}*b*d^{\gamma }$$

## 6. Simulations Results

### 6.1. Simulations of Security

**Figure 7:**
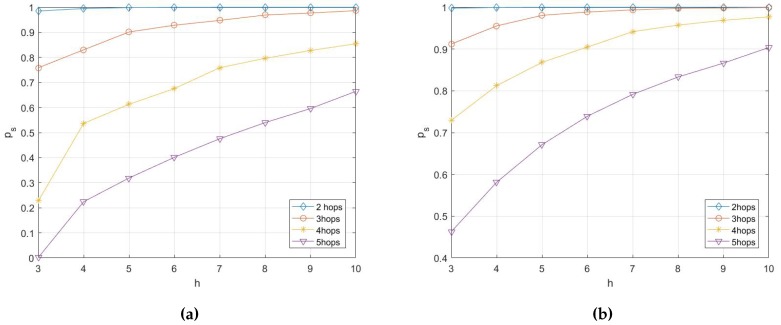
This is a figure that shows the relationship between the probability of transmission being secure and the multicast capacity with different $p_{o}$. (**a**) relationship between the probability of transmission being secure and capacity with $p_{o}=0.1$; (**b**) relationship between the probability of transmission being secure and capacity with $p_{o}=0.05$.

**Figure 8:**
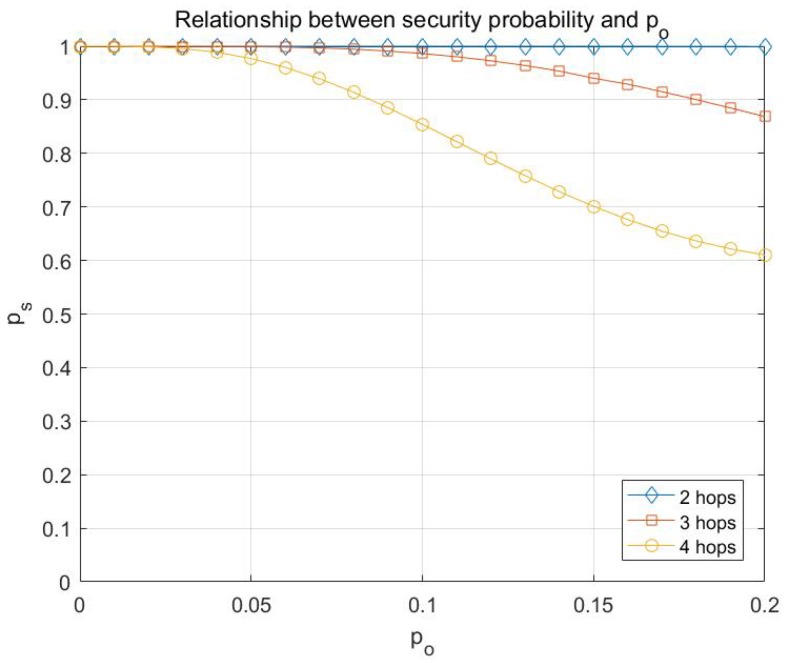
Relationship between the probability of transmission being secure $p_{s}$ and $p_{o}$ with multicast capacity is h=10.

**Figure 9:**
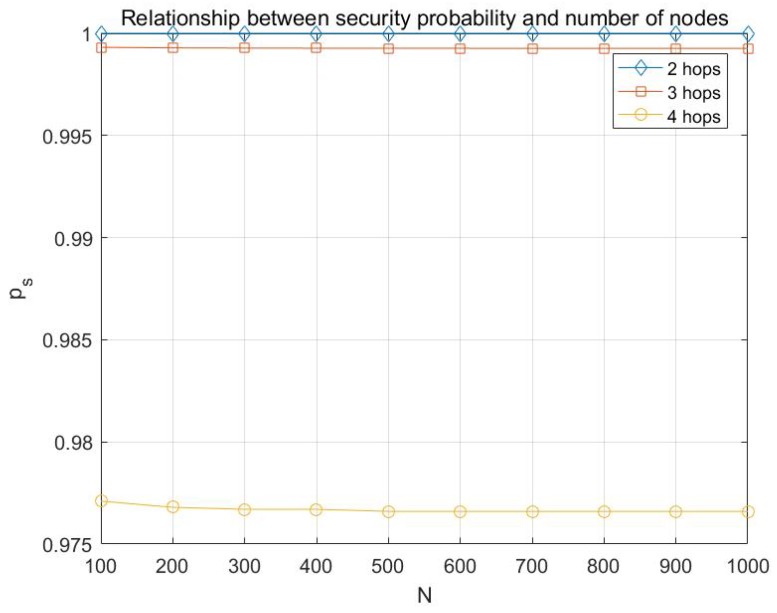
Relationship between the probability of transmission being secure $p_{s}$ and number of nodes with multicast capacity is h=10 and and $p_{o}=0.05$.

**Figure 10:**
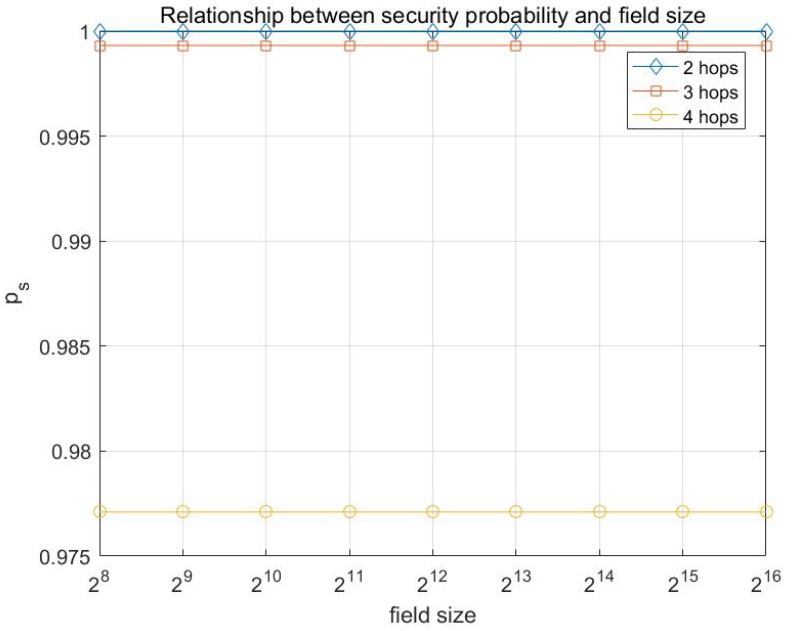
Relationship between the probability of transmission being secure $p_{s}$ and the size of the finite field with multicast capacity is h=10 and $p_{o}=0.05$.

Basing on the analysis in Section 4, the simulations on the probability of transmission being secure are conducted with different network parameters, including *h*, *e*, *k*, $p_{o}$, etc. Here the simulation results are presented in Figure 7, Figure 8, Figure 9 and Figure 10.

It can be concluded from Figure 7 that with the increase of multicast capacity *h*, the probability of transmission being secure $p_{s}$ increases slightly. However, With the increase of number of hops *k*, the probability of transmission being secure decreases rapidly. If the desired probability of transmission being secure is $p_{S}\geq 0.99$, the number of hops needs to be limited with k≤4.

From Figure 8, it can be concluded that the attacking capability of adversary has a relatively significant impact on the probability of transmission being secure, and the larger the number of hops is, the greater the impact is.

From Figure 9 and Figure 10, a conclusion can be drawn that the probability of transmission being secure is irrelevant with the number of nodes or the size of finite field.

### 6.2. Simulations of Energy Consumption

**Figure 11:**
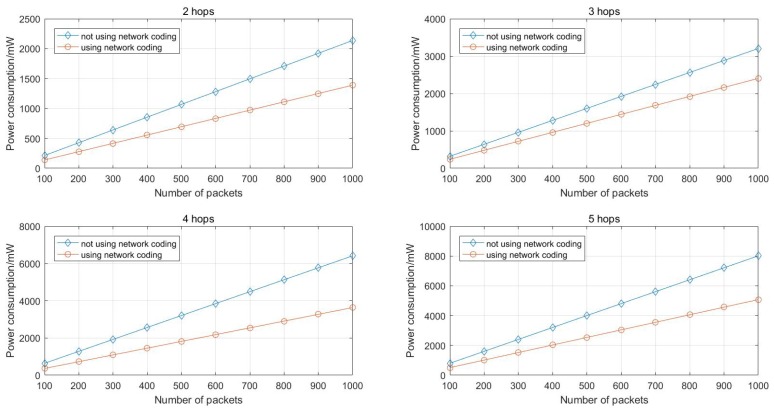
Energy consumed for transmitting $N_{p}=100:100:1000$ packets with different path length.

In Section 5, the analysis of energy consumption of the network coding based multipath routing scheme and the multipath routing scheme without network coding is conducted. Basing on that, simulations on energy consumption are performed and the results are presented in Figure 11.

As Figure 11 shows, it is clear that the network coding based scheme has a better energy efficiency and the more packets transmitted, the more energy consumption can be reduced.

## 7. Conclusions

In this article, a weakly secure network coding based multipath routing scheme is proposed. Based on that, the analysis on security and power consumption is conducted. Accordingly, the simulations on the probability of transmission being secure and power consumption are performed and the comparison of power consumption between two different schemes is performed as well. According to the analysis and simulation results, some conclusions can be drawn and are listed as follows:

1. As the number of hops of each path in the network increases, the probability of transmission being secure decreases rapidly, especially under the condition of low communication capacity. For example, when the the capacity is h=3 and $p_{o}=0.1$, with *k* being 2, 3, 4, and 5, the probability of transmission being secure is 0.9851, 0.7586, 0.2277, and 0.0020 correspondingly. Toward this end, if the desired probability of transmission being secure is $p_{s}\geq 0.99$, the number of hops should be limited with k≤4. To do this, it is necessary to deploy nodes with larger communication distance,especially when the nodes are deployed in a rather vast area.
2. When the number of hops k≥3, with the increase of multicast capacity *h*, the probability of transmission being secure increases and approaches 1 gradually. When k=2, with the increase of multicast capacity *h*, the probability of transmission being secure almost keeps unchanged and satisfies $p_{s}\approx 1$.
3. When the number of hops k≥3, the overhearing ability, which can be reflected by the figure of $p_{o}$, has a relatively significant impact on the probability of transmission being secure. However, when k=2, the probability of transmission being secure almost keeps unchanged and is approximately equal to 1.
4. Compared with the multipath routing scheme without network coding, the network coding based scheme in this article has a better energy efficiency. According to the simulation results, when 1000 packets are transmitted and the multicast capacity is h=10, the power consumption of network coding based multipath routing scheme is 36.67% less than the scheme without network coding.
